# Glass Fiber Reinforced Composite Orthodontic Retainer: In Vitro Effect of Tooth Brushing on the Surface Wear and Mechanical Properties

**DOI:** 10.3390/ma13051028

**Published:** 2020-02-25

**Authors:** Maria Francesca Sfondrini, Pekka Kalevi Vallittu, Lippo Veli Juhana Lassila, Annalisa Viola, Paola Gandini, Andrea Scribante

**Affiliations:** 1 Unit of Orthodontics and Paediatric Dentistry, Section of Dentistry, Department of Clinical, Surgical, Diagnostic and Paediatric Sciences, University of Pavia, 27100 Pavia, Italy; francesca.sfondrini@unipv.it (M.F.S.); annalisa.viola01@universitadipavia.it (A.V.); paola.gandini@unipv.it (P.G.); 2 Department of Biomaterial Science and Turku Clinical Biomaterials Centre (TCBC), Institute of Dentistry, University of Turku, 20100 Turku, Finland; pekval@utu.fi (P.K.V.); liplas@utu.fi (L.V.J.L.)

**Keywords:** FRC, bonding, technique, fiber, reinforced, composite, spot, mechanical, deflection, orthodontics, brushing, wear, retainer, splint, load

## Abstract

Fiber reinforced composites (FRCs) are metal free materials that have many applications in dentistry. In clinical orthodontics, they are used as retainers after active treatment in order to avoid relapse. However, although the modulus of the elasticity of FRCs is low, the rigidity of the material in the form of a relatively thick retainer with a surface cover of a flowable resin composite is known to have higher structural rigidity than stainless steel splints. The aim of the present study is to measure load and bending stress of stainless steel wires, as well as flowable resin composite covered and spot-bonded FRC retainer materials after tooth brushing. These materials were tested with a three point bending test for three different conditions: no brushing, 26 min of brushing, and 60 min of brushing. SEM images were taken before and after different times of tooth brushing. Results showed that stainless steel was not significantly affected by tooth brushing. On the other hand, a significant reduction of values at maximum load at fracture was reported for both FRC groups, and uncovered FRCs were most affected. Concerning maximum bending stress, no significant reduction by pretreatment conditions was reported for the materials tested. SEM images showed no evident wear for stainless steel. Flowable resin composite covered FRCs showed some signs of composite wear, whereas spot-bonded FRCs, i.e., without the surface cover of a flowable resin composite, showed signs of wear on the FRC and exposed glass fibers from the FRC’s polymer matrix. Because of the significant changes of the reduction of maximum load values and the wear for spot-bonded FRCs, this technique needs further in vitro and in vivo tests before it can be performed routinely in clinical practice.

## 1. Introduction

During the last years, fiber reinforced composites (FRCs) have been proposed for many clinical applications because they are easy to customize and manipulate, and they showed high improvements in properties if compared to unreinforced resins [1,2]. 

FRCs, if compared with other materials, have a high strength/weight and stiffness/weight ratio [3]. The most common fibers used in dentistry are glass fibers because of their low extensibility, high tensile strength, and aesthetic and optical qualities [4,5]. In dentistry, these materials are used for various purposes: fixed dental prostheses, fillings and core-built ups, removable devices, root canal anchoring systems, periodontal and trauma splints, orthodontic frameworks, and retainers [6,7,8,9,10,11,12,13]. 

Retention is an important phase of orthodontic treatment, especially because without any type of retention there is a tendency for the teeth to relapse [14]. Fixed retainers are the most common retention systems as they have a number of advantages. First of all, they provide better aesthetics, additional patient cooperation is not needed, and they are suitable for lifelong retention [4]. In the last 10 years, multi-stranded wires were the most popular type of retention in regards to bonded fixed retainers. At the same, time resin fiberglass bondings were introduced as an aesthetic alternative and they are widely used. Nowadays, there are many different types of retainers, removable and fixed, and it is unclear which ones are the best and for how long they should be used.

To our knowledge, there are no studies in literature about the prevalence of different bonding techniques, and each clinician uses the one that is more suitable according to its experience. There are only studies about the survival of different splinting techniques [9,15]. An extensive study of the literature suggests that there are significant variations in the results describing the effectiveness, cost factors, survival times, oral hygiene status, and regimen of various orthodontic retention appliances [16].

FRC retainers are composed of glass fibers, thermoplastic polymer, and light-cured resin matrix for the reinforcement of the dental polymer. [17,18]. Continuous unidirectional FRCs which are used in dental applications have a flexural modulus of 17 GPa and are influenced by absorption of water and volume fraction of fibers in the composite [19,20]. When glass FRC is used as an orthodontic retainer, the cross-sectional diameter of the retainer increases and this increases shear stresses within the FRC, as well as structural rigidity of the retainer. Some studies demonstrated that, if compared with metallic wires, FRC splints present high deflection values, showing mean indicative stiffness about 30 and 40 N under deflections of 1 and 2 mm with the average span length and cross-sectional dimension of an orthodontic retainer [21,22]. These values are higher than those for metallic wires, which are thinner in cross-sectional diameter. A high stiffness is useful in prosthodontics but is less desirable for splints and retainers as it can be in contrast with physiological tooth movement increasing the ankylosis risk, even if this concern has been tested in a single in vivo study, using an animal model [23]. Other studies demonstrated that the rigidity of a FRC splint is magnified by the FRC application technique [24,25].

In fact, following the manufacturer’s instructions, the composite covers the entire surface of the retainer. On the contrary, metallic splints are manufactured with a spot-bonding technique, thus allowing the wire to be covered with a composite only on the tooth surface, while the wire is left exposed in interproximal areas. The application of the spot bonding technique to FRCs implies a significant decrease in the rigidity of the framework if compared with a conventional full-bonded technique [24]. However, in this case, the fiber is exposed to the oral environment, thus leading to a higher wear risk, especially when the patient is eating or brushing their teeth. Wear is defined as the progressive loss of substance. It depends on the material type and geometry, on the interactions (stresses and forces), and on the environmental conditions (temperature, chemistry) [26]. 

Previous authors demonstrated that, increasing the number of brushing cycles, the abrasion of composite resins increased in a linear way [27]. There are no studies evaluating force levels of FRCs bonded with a spot technique simulating a different tooth brushing entity. Tooth brushing affects mechanical properties and wear of restorative materials. Authors evaluated its effects on a composite and we can suppose that FRC materials could probably also be affected, even if there is a lack of studies on this topic. The rationale of the present study was to test different FRC coverages for different variables.

The rationale of the present report is based on previous studies that demonstrated that FRC retainers have a higher rigidity if compared to metal splints and that this feature is less desirable. Previous studies also demonstrated that retainers made of FRCs without composite coverage have values of rigidity more similar to values of metal retainers, and this is a positive feature [21,22,24,25]. Finally brushing interferes with all restorative materials previously tested changing their mechanical properties [27]. Therefore, the data presented have the aim to test mechanical properties, maximum load and maximum bending stress, of covered and uncovered FRC, compare them to stainless steel, and evaluate eventual changing after tooth brushing. Additionally, their aim is to visually observe surfaces.

The purpose of this study was to evaluate mechanical properties (maximum bending stress and maximum load) and surface wear (SEM analysis) of metallic and FRC splints after various amounts of electrical tooth brushing, comparing the effects of tooth brushing on different kinds of materials. The null hypothesis of the present report was that no significant differences were reported in mechanical properties and wear among various materials tested. 

## 2. Materials and Methods 

In the present report, flat metallic splints (Straight 8 Lingual Retainer Wire 6′ length. DB Orthodontics, Silsden, United Kingdom), FRCs (Everstick ORTHO, StickTech, Turku, Finland) with composite coverage, and FRCs (Everstick ORTHO, StickTech, Turku, Finland) without composite coverage were tested. The fiber reinforced composite that was tested in the present study was a unidirectional FRC reinforced with silanized-treated glass fibers. This fiber-reinforced retainer contained 1000 silanized glass fibers plunged in a monomer-polymer gel matrix. With these materials, 72 specimens were prepared, 24 for each group. All specimens were cut with scissors to a size of 20 mm and handled accordingly to the manufacturer’s guidelines. 

### Metal Specimens Were Cut

Covered FRCs specimens were cut, covered with resin (Everstick Resin, GC America, Alsip, IL, USA), and subsequently light cured by hand using a halogen lamp (D-Light Pro, GC Europe, Leuven, Belgium) with a 1400 mW/cm^2^ wave length range of 430–480 nm for 40 s. Subsequently, the specimens were covered with a flowable particulate filler composite by hand (G-aenial Universal Injectable A2, GC America, Alsip, IL, USA) and light cured with the same halogen unit for 40 s [28]. The light tip of the curing unit was kept in 3 mm distance from the materials during light curing. Non-covered FRC specimens were prepared with the same technique without final flow composite coverage. All specimens were than stored in an incubator at 37 degrees Celsius for 24 h.

**Figure 1:**
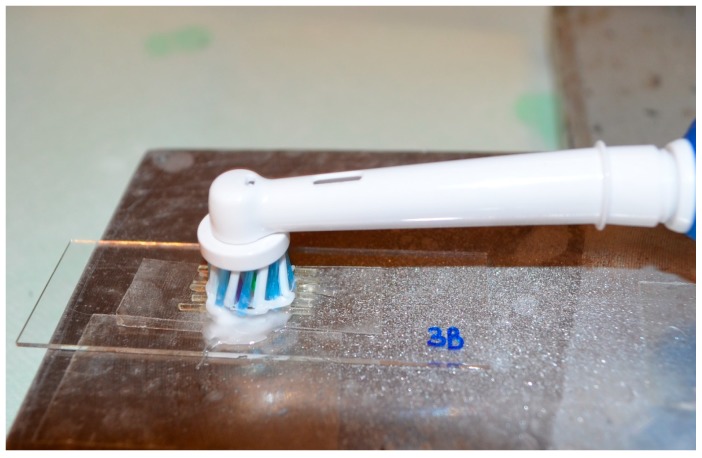
Brushing apparatus.

For each group (metal, covered FRC, non-covered FRC) 8 specimens were not brushed, 8 were subjected to electronic brushing for 26 min, and 8 were subjected to electronic brushing for 60 min. Brushed specimens were fixed at their edges on a laboratory glass (Figure 1) and brushed with an electric toothbrush (Oral B PRO 670 with Oral B Crossaction brush heads, Procter & Gamble, Cincinnati, USA) using a 124 RDA toothpaste (MaxWhite-white crystals, Colgate-Palmolive, New York, NY, USA).

The brushing set up was performed following a previous study [29]. Our aim was to attain contact between brush heads and specimens, which were posed on laboratory glasses and fixed by their extremity. Toothpaste was put on toothbrushes and toothbrushes were activated by pressing the start button. The chronometer was used in order to respect the time point.

Retainer material grouping was divided as follows:

(1) Flat Metal—Not brushed;
(2) Flat Metal—26 min brushed;
(3) Flat Metal—60 min brushed;
(4) Covered FRC—Not brushed;
(5) Covered FRC—26 min brushed;
(6) Covered FRC—60 min brushed;
(7) Non-covered FRC—Not brushed;
(8) Non-covered FRC—26 min brushed;
(9) Non-covered FRC—60 min brushed.

**Figure 2:**
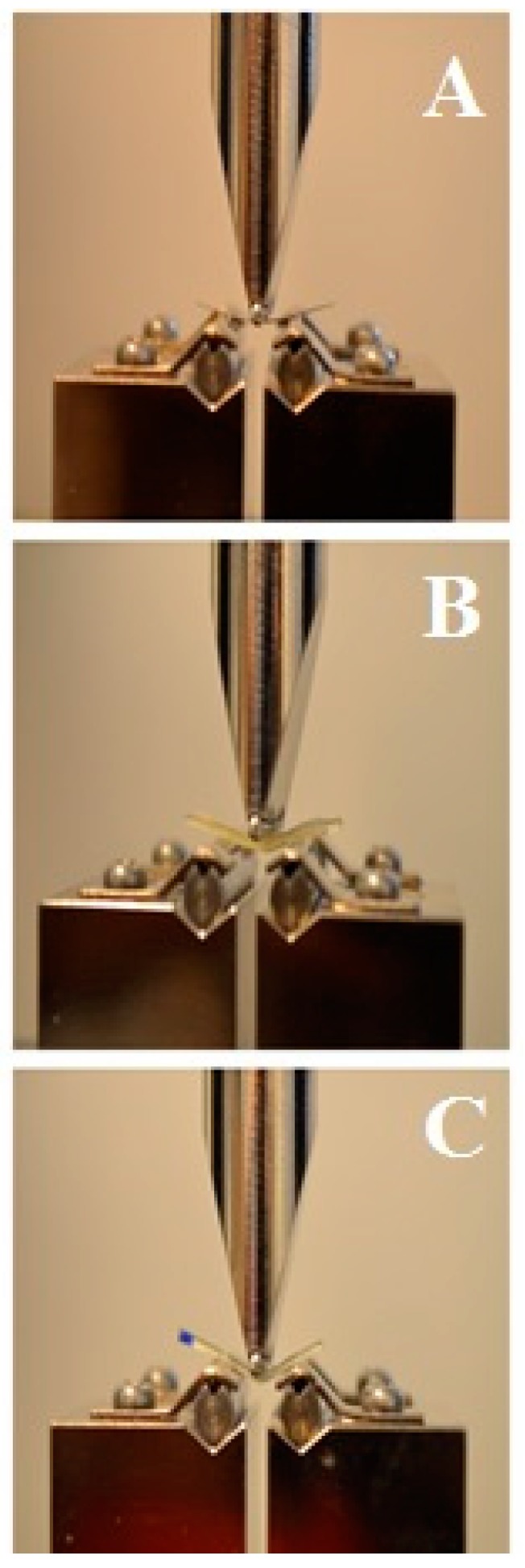
Three point bending test. (**A**) Metal wire, (**B**) fiber reinforced composite (FRC) full coverage, (**C**) FRC no coverage.

The retainer materials were subsequently evaluated with a three-point bending test (Figure 2). The span length was 14 mm, and the crosshead speed was 1 mm/min [30]. A universal testing machine (Lloyd LRX; Lloyd Instruments, Fareham, UK) was used to apply the load on the middle of the specimens tested. The middle point of the machine was moved using a computer-controlled stepper motor, the force was recorded by electronic sensors, and the position of the middle point of the machine was associated with the passive position. The flexural strength values were recorded with the Nexygen MT software (Lloyd Instruments) [31].

Maximum load (N) and maximum bending stress (MPa) were measured for each group [32]. Using a scanning electron microscope (JEOL 5500, JEOL Ltd., Tokyo, Japan), microphotographs for all the materials tested were taken with a magnification of 35×, 100×, and 250×. In order to use the scanning electron microscope, specimens were first submitted to sputter coating (BAL-TEC SCD050 Sputter Coater, Capovani Brothers Inc., New York, NY, USA).

Statistical analysis was performed with a computer software (R® version 3.1.3, R Development Core Team, R Foundation for Statistical Computing, Wien, Austria). For all groups, descriptive statistics, including mean, standard deviation, median, minimum, and maximum values, were calculated. The Kolmogorov–Smirnov test assessed normality of distributions. Analysis of variance (ANOVA) and post-hoc Tukey tests were used for inferential statistics. Significance was predetermined at *p* < 0.05 for all statistical tests.

## 3. Results

**Figure 3:**
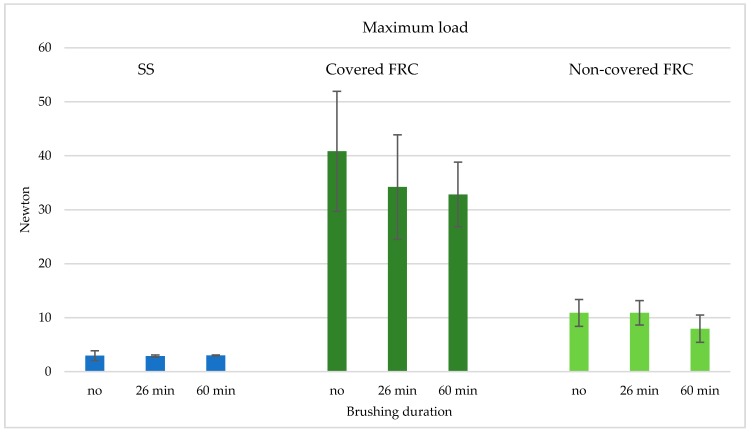
Graphical representation of maximum load values (Mean and SD) of the various conditions (metal, covered FRC, and non-covered FRC) after different brushing times (no brushing, 26 min of brushing, and 60 min of brushing).

The descriptive statistics for the maximum load evaluation values are listed in Table 1. Significant differences among various groups (*p* < 0.05) were demonstrated. The post-hoc Tukey test showed that the lowest values (*p* < 0.05) were reported with stainless steel wires (Groups 1, 2, and 3). The highest forces (*p* < 0.05) were demonstrated in FRCs bonded with a conventional covered technique (Groups 4, 5, and 6). When the experimental non-covered technique was tested (Groups 7, 8, and 9), intermediate measures were reported (Figure 3), with significantly higher values than the metal groups (*p* < 0.001) and significantly lower values than the covered groups (*p* < 0.001).

No difference was reported (*p* > 0.05) between not-brushed and brushed stainless steel groups (Groups 1–3). Conventional covered FRC groups (Groups 4–6) showed decreased values after 26 min of brushing (*p* < 0.05), and no differences between 26 and 60 min of brushing (*p* > 0.05). On the other hand, non-covered FRCs (Groups 7–9) showed a reduction only after 60 min of brushing (*p* < 0.05).

**Figure 4:**
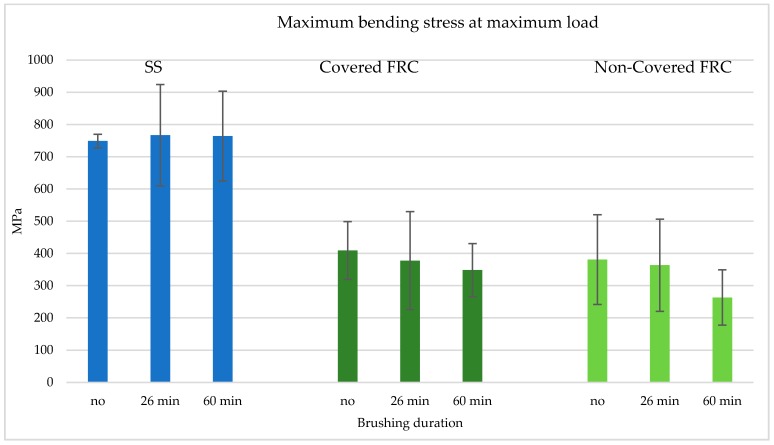
Graphical representation of maximum bending stress (Mean and SD) of the various conditions (metal, covered FRC, and non-covered FRC) after different brushing times (no brushing, 26 min of brushing, and 60 min of brushing).

Maximum bending stress results (Table 2) showed significant differences among the various groups tested (*p* < 0.05). Post-hoc analysis showed that the highest values (*p* < 0.05) were reported with stainless steel wires (Groups 1–3). Significantly lower values (*p* < 0.05) were reported for FRCs bonded with both conventional (Groups 4–6) and experimental (Groups 7–9) techniques (Figure 4) that showed no significant differences between them (*p* > 0.05). No significant difference was reported (*p* > 0.05) between not-brushed and brushed groups for all the three different conditions tested, even if there was a decrease of maximum bending stress values after tooth brushing in the FRCs groups.

## 4. Discussion

The null hypothesis of the present investigation was rejected: significant differences were reported among various groups. In this report, three different materials, which are used in orthodontic splints, were tested for different times of tooth brushing: stainless steel, FRC covered with a composite (used in the FRC covered conventional technique), and non-covered FRC (used in the experimental uncovered spot-bonding FRC technique) [24,25]. 

Rigidity (or bending stress) and maximum load are characteristics used to evaluate the longevity of retainers [21,22]. The load value in N with the predetermined magnitude of deflection was used as a descriptive value of the retainer´s rigidity. The highest values of maximum load were reported for FRC covered specimens (conventional technique), and these results confirm previous studies [21,22,29] which demonstrated the higher rigidity of fully covered FRC frameworks if compared to the metal ones. Uncovered FRCs showed lower values, as reported in previous studies [24,25]. The results of the present report are in agreement with previous investigations, showing that maximum load of uncovered FRC presented intermediate values between the conventional full covered FRC technique and metal splints.

Nowadays, there is only one in vitro study about the tooth brushing effect on these materials [30]. The previous study was made on Frasaco models splinted with different techniques in order to simulate a canine-to-canine splint. Mechanical (load at 0.1 mm deflection and at maximum load) and surface properties were tested. An experimental FRC spot bonding technique and metal splint technique seemed to have similar mechanical properties. 

However, tooth brushing is a daily routine for every patient, which is why further studies are needed before a clinical use of the experimental FRC technique. In the present report the same materials of the previous study were tested before and after different times of tooth brushing with a three point bending test (evaluating mechanical properties, such as maximum load and maximum bending stress) and with SEM microphotographs. 

Plaque is a predisposing factor to caries and periodontal disease, and dental hygiene is extremely important for oral health [33] as a soiled acidic environment could damage enamel [34], dentin [35], restorative materials [36], and prosthodontic frameworks [37]. In our research, a rotating oscillating electrical device was used, as rotation oscillation powered brushes significantly reduce plaque. The mechanical tooth brushing movement can lead to surface wear [38]. 

In dentistry, there are different kinds of wear: attrition, abrasion, and chemical wear. Concerning abrasive wear, material is scraped off the surface and this variable represents an important mechanism into oral environment [39,40]. Occlusal wear only concerns contact surfaces while tooth brushing abrasion can affect any exposed surface [41]. The abrasive composites wear was the subject of many studies and it is influenced by different factors, such as the size, shape, and the amount of filler, the resin matrix, and the bonding between the two phases [26]. In addition, the abrasive surface has an important role: its hardness is directly linked with the abrasion amount [42].

In the present test, the toothbrush used for all specimens was the Oral B PRO 670 toothbrush, Oral B crossaction brush heads (Procter & Gamble, Cincinnati, OH, USA). It was used in combination with a toothpaste (MaxWhite-white crystals, Colgate-Palmolive, New York, NY, USA) with a RDA (Relative Dentin Abrasion) of 124. Our aim was to analyze how mechanical properties and surface wear of different materials change with the same abrasive. Further studies can be made by testing the abrasion of different brands of toothbrushes and toothpastes, evaluating if their different abrasive power will produce different effects in mechanical properties and surface wear of FRC splints. 

As previous authors reported on in vitro brushing test [29], the present test was performed as a result of a simulated six-month long tooth brushing period, supposing that patients brush their teeth everyday with an average of twice a day during 6 months. An average brushing time of 2 min for each time was considered, two times per day. Next, the result was divided by 28, the expected number of teeth, in order to find the mean brushing time of each single tooth. The result was 26 min, thus specimens were experimentally brushed for this amount of time. Moreover, in the present report, the same times of tooth brushing were applied. Six months (26 min of simulated continuous brushing) and 14 months (60 min of simulated continuous brushing) were tested.

Additionally, the three point bending test was conducted after the specimens were stored in an incubator at 37 degrees Celsius for 24 h. It would be interesting to perform further tests after subjecting the glass reinforced composite into artificial saliva, in order to ascertain if oral lubrification could influence surface wear.

In the present report, mechanical properties were evaluated with a three point bending test, no cyclical loading test was performed. A bending test would relate more directly to orthodontic retention and relapse with a cyclical loading test, so further studies would be welcomed in order to test this additional variable.

Stainless steel is the most common material used for orthodontic retainers, and it is well accepted by patients [43,44,45]. Only one in vitro study [29] tested orthodontic stainless steel wires after tooth brushing, showing no significant differences in mechanical properties over time. No three point bending tests were performed after tooth brushing. 

In this report stainless steel specimens were tested before tooth brushing, after 26 min, and after 60 min of tooth brushing: no significant difference was shown both for maximum load and for maximum bending stress. Conversely, a significant reduction of maximum load values was reported in a previous study [29], but the reduction of values after tooth brushing were due to the wear composite used to fix the wires. Therefore, tooth brushing does not damage metal wires. 

Concerning FRC with coverage, after tooth brushing, a significant decrease of maximum load values was reported after 26 min. After 60 min of tooth brushing, no significant difference was reported. Instead, for the maximum bending stress at maximum load, there was no significant reduction of values after tooth brushing. A previous report tested maximum load of conventional FRCs after 26 min of brushing, showing no significant differences. The variability of the results is probably due to the different investigation methods (Frasaco models and three point bending tests).

In the present study, FRC without coverage after tooth brushing presented a significant decrease of maximum load values after 60 min, but no significant decrease between 0 and 26 min. No significant reduction of maximum bending stress was reported before and after tooth brushing.

Concerning the differences between metal and FRCs behavior, they seem to be ascribed to the intrinsic differences of the two materials tested and to their different responses into a three point bending test. Concerning maximum bending stress, stainless steel wires showed no differences after brushing. On the other hand, both FRC groups showed a decrease in mean values, but the decrease was not significant. Further tests should be conducted in the future analyzing FRC maximum bending stress behavior after longer brushing times in order to determine if the decrease will be significant with more brushing time.

**Figure 5:**
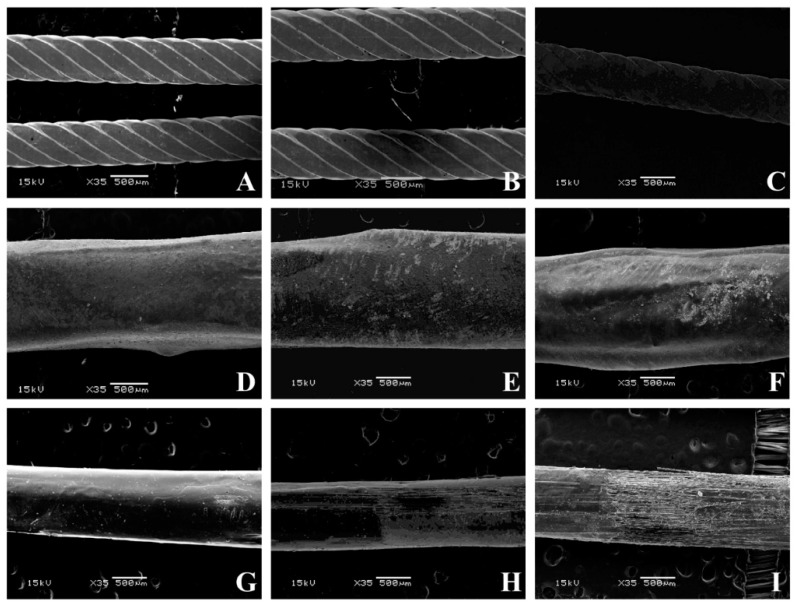
SEM pictures of various conditions tested at 35× magnification. (**A**) Flat metallic wire not brushed, (**B**) flat metallic wire brushed for 26 min, (**C**) flat metallic wire brushed for 60 min, (**D**) full-bonded FRC not brushed, (**E**) full-bonded FRC brushed for 26 min, (**F**) full-bonded FRC brushed for 60 min, (**G**) spot-bonded FRC not brushed, (**H**) full-bonded FRC brushed for 26 min, (**I**) full-bonded FRC brushed for 60 min.

**Figure 6:**
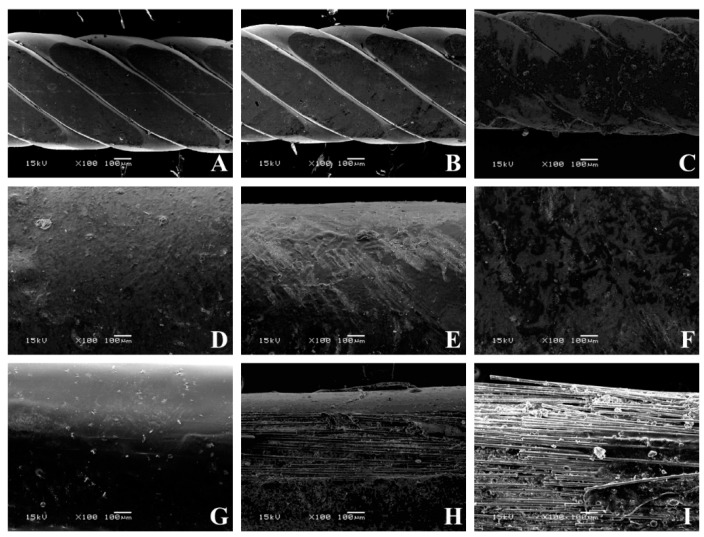
SEM pictures of various conditions tested at 100× magnification. (**A**) Flat metallic wire not brushed, (**B**) flat metallic wire brushed for 26 min, (**C**) flat metallic wire brushed for 60 min, (**D**) full-bonded FRC not brushed, (**E**) full-bonded FRC brushed for 26 min (**F**) full-bonded FRC brushed for 60 min, (**G**) spot-bonded FRC not brushed, (**H**) full-bonded FRC brushed for 26 min, (**I**) full-bonded FRC brushed for 60 min.

**Figure 7:**
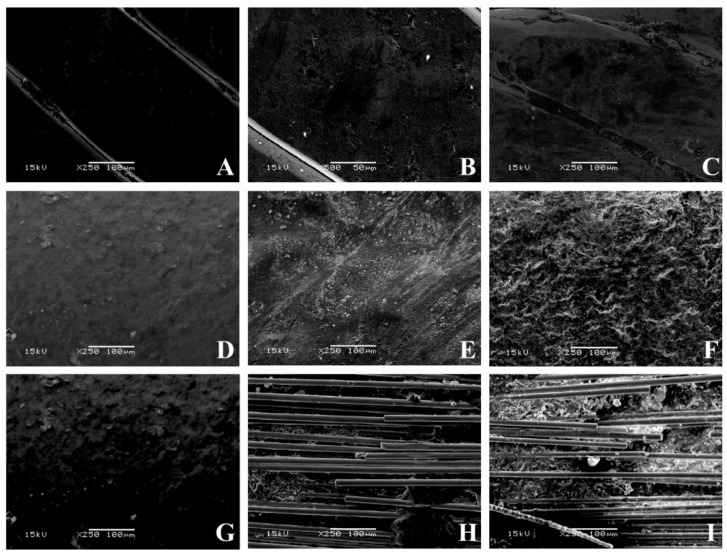
SEM pictures of various conditions tested at 250× magnification. (**A**) Flat metallic wire not brushed (**B**) flat metallic wire brushed for 26 min, (**C**) flat metallic wire brushed for 60 min, (**D**) full-bonded FRC not brushed, (**E**) full-bonded FRC brushed for 26 min, (**F**) full-bonded FRC brushed for 60 min, (**G**) spot-bonded FRC not brushed, (**H**) full-bonded FRC brushed for 26 min, (**I**) full-bonded FRC brushed for 60 min.

Pictures of all specimens with a magnification of 35× (Figure 5), 100× (Figure 6), and 250× (Figure 7) were taken with a scanning electron microscope. SEM images provided a qualitative, not quantitative, evaluation, which is a limitation of the test. However, SEM images showed visual signs of wear. No visual signs of wear were reported on stainless steel specimens, both after twenty-six minutes and one hour of tooth brushing. Common signs of composite wear were reported on FRC covered with a composite, similar to those reported in other studies [29,46,47,48]. These scratches are more evident after one hour of tooth brushing than after 26 min of tooth brushing. Remarkable signs of wear were reported on FRC left uncovered, and higher after 60 min with disarranged and broken fibers. All dental materials are subject to wear. Wear can reduce the resistance of the material, change its mechanical and aesthetic properties, and lead to bacterial adhesion [46,47].

Results on FRCs without coverage after 26 min of tooth brushing are in agreement with a previous study in which microphotographs were taken with a magnification of 35× and 100× [29]. No studies have been conducted on the FRC surface after 1-h tooth brushing, thus no direct comparison can be made on wear sign results. Before a clinical use of this experimental spot-bonded FRC technique, further in vitro and in vivo tests are needed to evaluate other variables of fibers left uncovered, such as bonding efficiency, duration, and bacterial adhesion. 

Limitations of the present study are related to the materials used. In fact, only some materials have been taken into consideration, even if other FRC materials are nowadays present on the market with different shapes, sizes, and diameters of fibers. Different geometries of materials tested could influence results. Moreover, our in vitro study cannot completely simulate real clinical conditions. In fact, even though many in vitro studies have been conducted, the main limitation of a FRC clinical use is that research is still lacking regarding long-term clinical performance [49]. As wear, delamination and fracture of FRC devices have been reported [48]. In order to confirm the results of the present investigation, which is a pilot study, randomized controlled clinical trials would be welcomed.

## 5. Conclusions

The present study demonstrated that after tooth brushing no mechanical differences were reported for metal specimens, while a reduction of maximum load values was reported for all FRC specimens. SEM images showed that after tooth brushing metal specimens were not visually affected, while signs of wear were reported for all FRC groups, especially for specimens not covered with a composite. 

## Figures and Tables

**Table 1:** Descriptive statistics of the different groups at maximum load (N).

Group	Material	Coverage	Brushing	Mean	St Dev	Min	Mdn	Max	Significance *
1	SS	no	no	2.98	0.91	2.23	2.38	4.17	A
2	SS	no	26 min	2.91	0.21	2.40	2.99	3.00	A
3	SS	no	60 min	3.02	0.08	2.90	3.08	3.08	A
4	FRC	full	no	40.82	11.13	26.58	40.15	60.30	B
5	FRC	full	26 min	34.20	9.67	23.17	32.14	48.07	C
6	FRC	full	60 min	32.84	5.99	24.30	32.46	40.91	C
7	FRC	no	no	10.89	2.49	8.85	9.57	15.22	E
8	FRC	no	26 min	10.90	2.26	7.24	11.22	14.19	E
9	FRC	no	60 min	7.97	2.54	5.54	7.43	12.59	F

* Means with the same letters are not significantly different (*p* > 0.05).

**Table 2:** Descriptive statistics (MPa) of the maximum bending stress of the different groups.

Group	Material	Coverage	Brushing	Mean	St Dev	Min	Mdn	Max	Significance *
1	SS	no	no	748.61	21.14	712.33	750.36	777.77	A
2	SS	no	26 min	766.88	157.10	546.00	784.00	967.00	A
3	SS	no	60 min	764.26	139.16	578.72	759.55	957.00	A
4	FRC	full	no	409.35	89.70	275.07	392.21	571.71	B
5	FRC	full	26 min	377.65	151.78	219.73	315.24	626.15	B
6	FRC	full	60 min	348.25	82.53	225.39	343.33	442.79	B
7	FRC	no	no	381.03	139.23	205.57	337.30	612.25	B
8	FRC	no	26 min	363.61	143.04	208.41	318.67	588.38	B
9	FRC	no	60 min	263.32	85.64	181.18	245.61	454.61	B

* Means with the same letters are not significantly different (*p* > 0.05).
